# Suppression of miR-708 inhibits the Wnt/β-catenin signaling pathway by activating DKK3 in adult B-all

**DOI:** 10.18632/oncotarget.19342

**Published:** 2017-07-18

**Authors:** Yingjie Zhang, Huibo Li, Rongyi Cao, Lili Sun, Yan Wang, Shengjin Fan, Yanqiu Zhao, Desheng Kong, Lin Cui, Leilei Lin, Ke Wang, Yinghua Li, Jin Zhou

**Affiliations:** ^1^ Department of Hematology, The First Affiliated Hospital, Harbin Medical University, Harbin, China; ^2^ Heilongjiang Academy of Medical Science, Harbin, China

**Keywords:** adult B-acute lymphoblastic leukemia, miR-708, 5-aza-2’-deoxycytidine, DKK3, Wnt/β-catenin

## Abstract

Inactivation of Dickkopf-3 (DKK3) is closely associated with a poor prognosis in various solid tumor and hematologic malignancies. Promoter hypermethylation is one potential cause of DKK3 inactivation. However, whether other mechanisms lead to DKK3 inactivation and the subsequent effects of these inactivations on cell proliferation and the Wnt signaling pathway in adult B acute lymphoblastic leukemia (B-ALL) remain unclear. In the present study, we found that low DKK3 expression levels were associated with high miR-708 expression and promoter hypermethylation in adult B-ALL. miR-708 was confirmed to directly decrease DKK3 expression in Nalm-6 and BALL-1 cells. Additionally, a miR-708 inhibitor decreased cell proliferation mainly through apoptosis and cell cycle arrest at the G1 phase, and these effects were eliminated by DKK3 siRNA treatment. Moreover, the demethylating agent 5-aza-2′-deoxycytidine (5-aza) decreased the methylation state of the DKK3 promoter based on methylation-specific PCR (MSP) and bisulfite genomic sequencing PCR (BSP), although this demethylation effect was not enhanced by the miR-708 inhibitor. The miR-708 inhibitor or 5-aza significantly increased DKK3 expression and decreased p-GSK3β, cyclin D1 and nuclear and cytoplasmic β-catenin protein expression, indicating that the Wnt/β-catenin signaling pathway was inhibited. These effects became more pronounced when the miR-708 inhibitor and 5-aza were used simultaneously. These findings provide greater insights into the mechanisms that increase DKK3 expression and suggest that a miR-708 inhibitor and 5-aza might be useful as targeted therapies for adult B-ALL.

## INTRODUCTION

Adult B acute lymphoblastic leukemia (B-ALL) has a poor prognosis, with a 5-year overall survival of approximately 35% [1]. Although treatments have notably improved over the past decade, the outcomes in adults remain unfavorable due to high chemoresistance or relapse [2, 3]. Revealing the intricate regulatory networks that exist among important molecules and signaling pathways might provide insights into this disease and suggest new strategies for treatment.

Human Dickkopf-3 (DKK3) is a member of the Dickkopf family, which is a group of secreted glycoproteins known to inhibit the Wnt signaling pathway [4]. The downstream effector of Wnt signaling is nuclear non-phosphorylated β-catenin, which acts in association with the LEF/TCF (lymphoid enhancer factor/T cell factor) family of transcription factors to mediate the expression of several genes, including cyclin D1 and c-Myc [5]. The silencing of Wnt antagonist genes is associated with activation of the Wnt signaling pathway and is regarded as an independent poor prognostic factor for patients with acute myelocytic leukemia (AML) [6]. As an antagonist of the Wnt signaling pathway, decreased DKK3 expression is also related to dismal prognoses in breast cancer, renal cell carcinoma and ALL patients [7–11]. Down-regulation or silencing of DKK3 is associated with promoter CpG methylation in chronic lymphocytic leukemia, myelodysplastic syndrome, AML and ALL [9–14]. In addition to DNA methylation, microRNAs (miRNAs), which are also epigenetic regulators that can inactivate important molecules, have been extensively explored in the pathogenesis of hematologic malignancies. In solid tumors, DKK3 is regulated by miRNAs, such as miR-92b and miR-18 [15, 16]. However, the regulation and biological functions of DKK3 in adult B-ALL remain unclear.

miRNAs play key roles in the regulation of gene expression, and their dysregulation is associated with the pathogenesis of various solid cancers and hematologic malignancies [17, 18]. Due to their tissue- and disease-specific expression patterns and their tremendous regulatory potential, miRNAs can function as oncomirs or tumor suppressors in different types of cancers, depending on their target mRNAs [19]. miR-708 promotes cell survival in colorectal cancer by targeting cyclin-dependent kinase inhibitor 2B [20], whereas this miRNA decreases cell growth, clonality, invasion and migration in renal cell carcinoma, breast cancer and hepatocellular carcinoma [21–23]. In childhood ALL, miR-708 is related to a high risk of relapse and a good glucocorticoid response [24, 25]. Nonetheless, the precise roles of miR-708 in the pathogenesis and mechanisms underlying adult B-ALL are unclear.

In this study, we detected low DKK3 expression in adult B-ALL patients and cell lines. This low expression was mainly due to promoter hypermethylation and high miR-708 expression. We revealed that miR-708 directly targets DKK3 and causes B-ALL cell proliferation through cell cycle promotion and apoptosis inhibition. The results showed that suppression of miR-708 expression and/or treatment with 5-aza-2′-deoxycytidine (5-aza) increases DKK3 expression and inhibits the Wnt/β-catenin signaling pathway. These findings provide a novel strategy for the treatment of adult B-ALL.

## RESULTS

### DKK3 and miR-708 expression in B-ALL patients and cell lines

**Figure 1:**
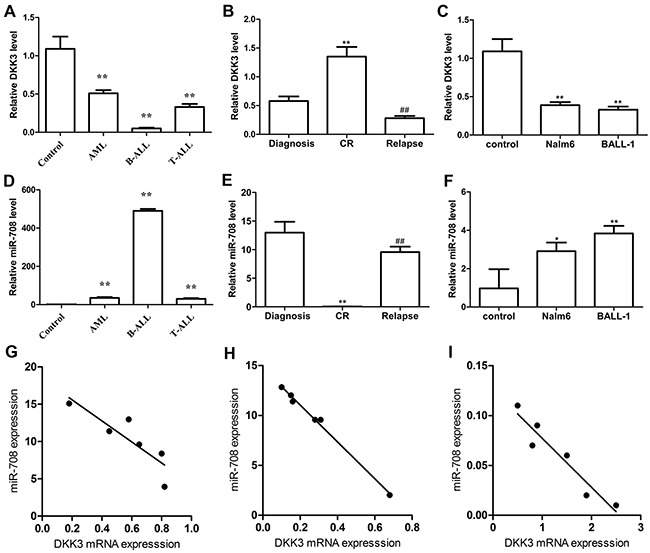
DKK3 mRNA and miR-708 expression levels in acute leukemia patients and cell lines evaluated by PCR **(A** and **D)** Compared with the controls (monocytes (MNCs) from healthy volunteers, n=3), DKK3 mRNA in newly diagnosed AML (n=10), B-ALL (n=20) and T-ALL (n=7) patients was expressed at low levels (A), and miR-708 was highly expressed (D). **(B** and **E)** The DKK3 mRNA expression level was low (B) and miR-708 was highly expressed (E) in newly diagnosed and relapsed B-ALL patients (n=6) compared with samples extracted at complete remission (CR). **(C** and **F)** Compared with the control (MNCs from healthy volunteers), DKK3 mRNA was expressed at low levels (C) and miR-708 was highly expressed (F) in the B-ALL cell lines. **(G-I)**. The miR-708 and DKK3 mRNA expression levels are negatively correlated in newly diagnosed (G), relapsed (H) and CR (I) B-ALL patients. The data are presented as the means ± standard deviations (SDs) of three different experiments. *P<0.05; ** and^##^ *P*<0.01.

The tumor suppressor gene DKK3 is expressed at low levels in many solid tumors and hematologic malignancies [7–11]. We measured the DKK3 mRNA expression levels in patients with acute leukemia by PCR. Compared with monocytes from healthy volunteers, DKK3 expression in patients with AML, B-ALL and T-ALL was reduced by 0.48-, 0.046- and 0.33-fold, respectively (Figure 1A). Among these patients, DKK3 was expressed at the lowest level in the B-ALL patients. To determine whether DKK3 expression was correlated with clinical features, we measured the DKK3 mRNA expression levels at diagnosis, complete remission (CR) and relapse in the same patients with B-ALL (P13, 14, 16 and, 28-30). Figure 1B shows that DKK3 expression was markedly lower in the paired initial diagnosis and relapse samples than in the matched CR samples. We also detected low DKK3 mRNA expression in two B-ALL cell lines (Nalm-6 and BALL-1; Figure 1C).

We used the miRanda and miRBase algorithms to predict miRNA species that directly targeted DKK3. Among the predicted miRNAs, miR-708 attracted our attention because its expression was correlated with the prognosis of ALL in children [24, 25], although its role in adult B-ALL remained unclear. Compared with monocytes (MNCs) from the healthy volunteers, miR-708 expression in patients with AML, B-ALL and T-ALL was increased by 35.07-, 490.2-, and 30.3-fold, respectively (Figure 1D). Additionally, the miR-708 expression level was higher in newly diagnosed and relapsed patients than in samples collected from the same patients when they achieved CR (Figure 1E), suggesting that miR-708 overexpression might be related to the prognosis in adult B-ALL patients. miR-708 expression was also high in Nalm-6 and BALL-1 cells compared with MNCs from healthy volunteers (Figure 1F). These findings showed that miR-708 and DKK3 had opposite expression patterns in adult B-ALL patients and cell lines. A Spearman's correlation analysis showed that DKK3 mRNA expression was negatively correlated with miR-708 expression in adult B-ALL patients who were newly diagnosed, undergoing CR and relapsed (Figures 1G-1I).

### miR-708 directly targets the 3′-UTR of DKK3

**Figure 2:**
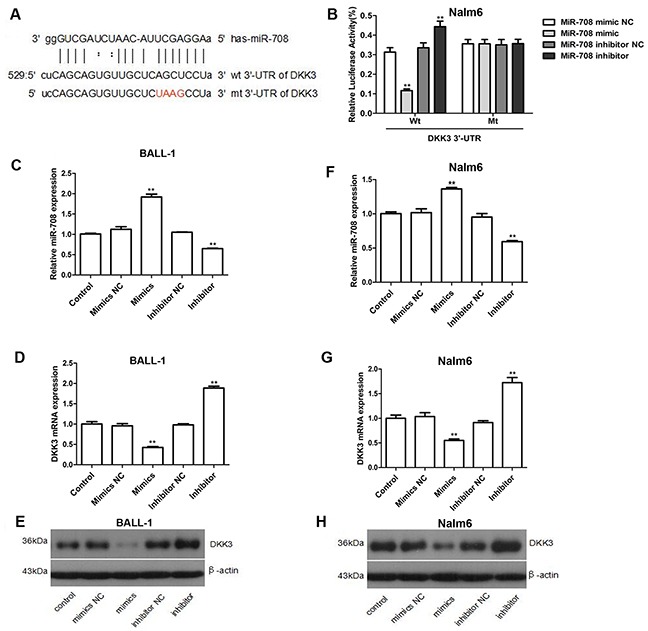
DKK3 is a functional target of miR-708 in B-ALL **(A)** Predicted binding sites for miR-708 and DKK3. The sequence of the mutant 3′-UTR of DKK3 is also presented. **(B)** Luciferase assays showing the decrease in relative luciferase activity in Nalm-6 cells co-transfected with miR-708 and DKK3 and the increase in cells co-transfected with a miR-708 inhibitor and DKK3. A mutated 3′-UTR DKK3 plasmid was used as the control. **(C-H)**. The miR-708 (C and F) and DKK3 mRNA levels (D and G) and the DKK3 protein expression levels (E and H) changed after transfection with a miR-708 mimics or a miR-708 inhibitor, respectively. (D and G). The graphs show the fold changes in protein expression relative to the untreated control measured by densitometry. (E and H) The images represent the results of a western blotting analysis of DKK3 protein expression. The data are presented as the means ± SDs of three different experiments. NC indicates the negative control. *P<0.05; **P<0.01.

The relationship between miR-708 and DKK3 was subsequently demonstrated by luciferase activity. Because miR-708 was predicted to bind to the 3′-untranslated region (UTR) of DKK3 by miRanda (Figure 2A), we constructed firefly luciferase reporters containing either the entire wild-type or a mutant version of the DKK3 3′-UTR. Co-transfection of miR-708 and the luciferase reporter with the wild-type DKK3 3′-UTR greatly reduced the luciferase activity (Figure 2B), whereas application of a miR-708 inhibitor eliminated this inhibition and strongly increased the relative luciferase activity. Conversely, no significant difference was found for DKK3 luciferase activity in Nalm-6 cells co-transfected with the mutant DKK3 3′-UTR. We then measured the DKK3 mRNA and protein expression levels in cells transfected with either a miR-708 mimics or a miR-708 inhibitor. The miR-708 mimics significantly decreased the DKK3 mRNA and protein levels in BALL-1 and Nalm-6 cells, whereas the miR-708 inhibitor markedly increased DKK3 expression in these cells (Figures 2C-2H). These data indicate that miR-708 directly binds to the 3′-UTR of DKK3 and significantly down-regulates its mRNA and protein expression.

### Suppression of miR-708 expression inhibits cell proliferation

**Figure 3:**
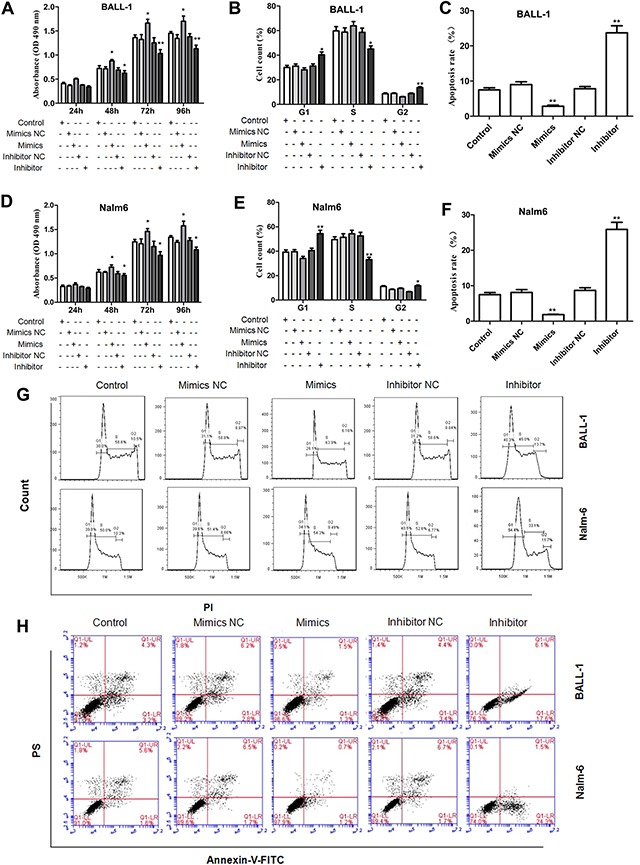
Influence of miR-708 expression on cell proliferation, the cell cycle and apoptosis **(A, D)**. A miR-708 inhibitor significantly decreased the proliferation rates of BALL-1 (A) and Nalm-6 (D) cells. **(B** and **E)** The miR-708 inhibitor significantly increased the proportions of BALL-1 (B) and Nalm-6 (E) cells in the G1 phase. **(C, F)** The miR-708 inhibitor increased the numbers of apoptotic BALL-1 (C) and Nalm-6 (F) cells. **(G** and **H)**. Representative depictions of the cell cycle (G) and apoptosis (H) in the two cell lines. Non-transfected cells were used as controls. Three independent experiments were performed. The data are presented as the means ± SDs. *P<0.05; **P<0.01.

Subsequently, we evaluated the effect of miR-708 on the proliferation of the B-ALL cell lines. As shown in Figure 3A and 3D, the miR-708 inhibitor inhibited cell proliferation, whereas the miR-708 mimics promoted proliferation of the BALL-1 and Nalm-6 cells. Cell cycle progression and apoptosis were examined to explore the mechanism(s) through which miR-708 suppression inhibits ALL cell growth. After treatment of the cells with the miR-708 inhibitor, the proportion of BALL-1 cells in G1 increased from 30.6% to 40.3%, and the proportion of Nalm-6 cells in G1 increased from 39.3% to 54.4%. Treatment with the miR-708 inhibitor decreased the proportion of BALL-1 cells in S from 59.8% to 45% and the proportion of Nalm-6 cells from 49.5% to 33.1% (Figures 3B, 3E and 3G). However, the miR-708 mimics did not affect the proportion of cells in the S and G2 phases. Additionally, the number of apoptotic cells increased in response to the miR-708 inhibitor but decreased in response to the miR-708 mimics in both cell lines (Figures 3C, 3F and 3H). These results indicate that suppression of miR-708 inhibits the growth of B-ALL cell lines by arresting the cells in the G1 phase and increasing apoptosis.

**Figure 4:**
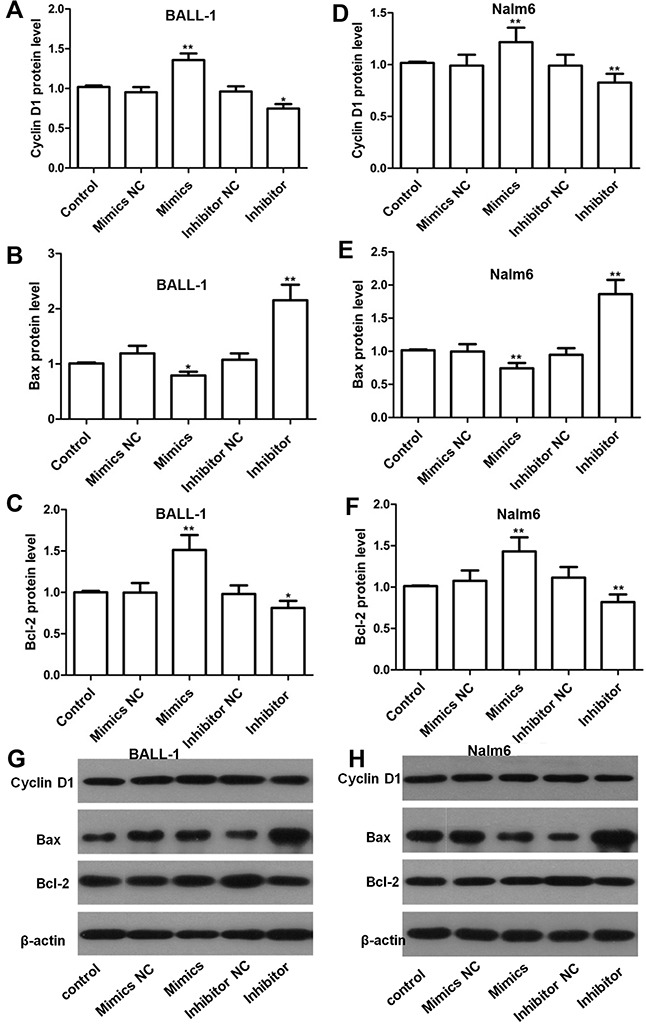
Effects of miR-708 on the expression of proteins related to the cell cycle and apoptosis as determined by western blotting **(A** and **D)**. The miR-708 inhibitor decreased cyclin D1 expression in BALL-1 (A) and Nalm-6 (D) cells. **(B-F)** The miR-708 inhibitor significantly increased Bax expression and strongly decreased Bcl-2 expression in BALL-1 (B and C) and Nalm-6 cells (E and F). Non-transfected cells were used as controls. The fold changes in the relative protein levels were calculated with reference to the control levels. The data are presented as the means ± SDs (n=3, * *P*<0.05; ** *P*<0.01). **(G** and **H)**. The images represent the results of a western blotting analysis of cyclin D1, Bax and Bcl-2 protein expression.

To elucidate the mechanism through which the miR-708 inhibitor regulated the cell cycle and apoptosis, we examined relevant proteins by western blotting. As shown in Figure 4(Figures 4A, 4D, 4G and 4H), the level of cyclin D1, which is a key regulator of the G1/S checkpoint, was reduced by the miR-708 inhibitor in both BALL-1 and Nalm-6 cells. These results correlate with the cell cycle arrest produced by the miR-708 inhibitor. Additionally, the suppression of miR-708 expression led to increased Bax levels and decreased Bcl-2 levels in both cell lines, whereas miR-708 overexpression reduced Bax and increased Bcl-2 expression in these cells (Figures 4B, 4C, 4E-4H). These data are in agreement with the observed apoptosis caused by the miR-708 inhibitor. Suppression of miR-708 inhibited cell proliferation via both cell cycle arrest at the G1 phase and the promotion of apoptosis.

### Depletion of DKK3 attenuates the effects of the miR-708 inhibitor on cell proliferation

**Figure 5:**
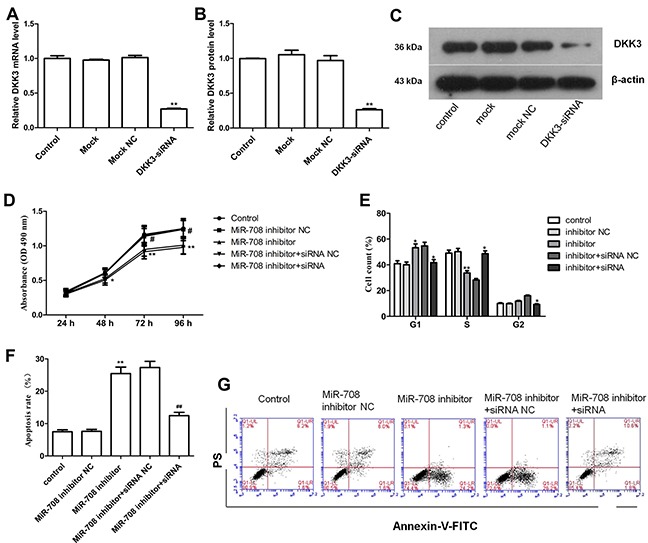
Depletion of DKK3 by siRNA eliminates the effect of a miR-708 inhibitor on Nalm-6 cells **(A-C)**. DKK3 mRNA (A) and protein (B and C) expression was significantly inhibited by the DKK3 siRNA, as determined by qRT-PCR and western blotting. The fold changes are relative to the untreated controls. **(D)** The DKK3 siRNA significantly increased cell proliferation in Nalm-6 cells, even in the presence of the miR-708 inhibitor. **(E)** The DKK3 siRNA strongly decreased the proportion of Nalm-6 cells in the G1 phase and increased the proportion of cells in the S phase, even in the presence of the miR-708 inhibitor. **(F)** The DKK3 siRNA markedly decreased apoptosis, even in the presence of the miR-708 inhibitor. **(G)**. Representative plots of apoptosis in the cell lines. The data are presented as the means ± SDs of three different experiments. The miR-708 inhibitor NC was used as a control for the miR-708 inhibitor. The miR-708 inhibitor and the siRNA NC were used as controls for the miR-708 inhibitor and DKK3 siRNA, respectively. # and * means *P*<0.05; ** and ## means *P*<0.01.

After showing that miR-708 significantly inhibits the luciferase activity by interacting with the DKK3 3′-UTR, we sought to determine whether DKK3 is a functionally important target of miR-708 in Nalm-6 cells. DKK3 mRNA and protein expression was effectively reduced by transfection of the Nalm-6 cells with the DKK3 siRNA (Figures 5A-5C), and this depletion of DKK3 alleviated the inhibition of cell proliferation by the miR-708 inhibitor (Figure 5D). The proportion of Nalm-6 cells in the G1 phase was also increased by the miR-708 inhibitor and decreased by the DKK3 siRNA when both were present (Figure 5E). Additionally, the total numbers of early and late apoptotic cells were increased by the miR-708 inhibitor but decreased by the DKK3 siRNA (particularly the late apoptotic cells), even when the miR-708 inhibitor was present (Figure 5F and 5G). Overall, these results indicate that DKK3 is functionally important for the miR-708 inhibition-mediated regulation of cell proliferation.

### The miR-708 inhibitor and 5-aza increase DKK3 expression through two independent mechanisms

**Figure 6:**
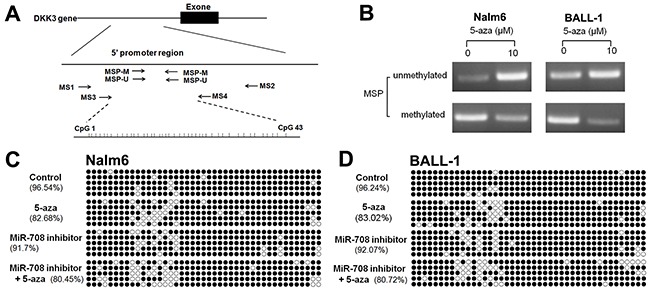
Effects of the miR-708 inhibitor and/or 5-aza on the methylation state of the DKK3 promoter **(A)** Genomic structure of the DKK3 gene and locations of the primers used in this study. **(B)** Detection of the unmethylated and methylated statuses of the DKK3 gene using the MSP method. **(C and D)** Methylation status of the DKK3 gene promoter region in CpG islands in Nalm- 6 (C) and BALL-1 (D) cells treated with the miR-708 inhibitor and/or 5-aza, as determined by sequencing after bisulfite modification of the genomic DNA. The solid spots means methylated CpG dinucleotide, the hollow spots means unmethylated CpG dinucleotide.

**Figure 7:**
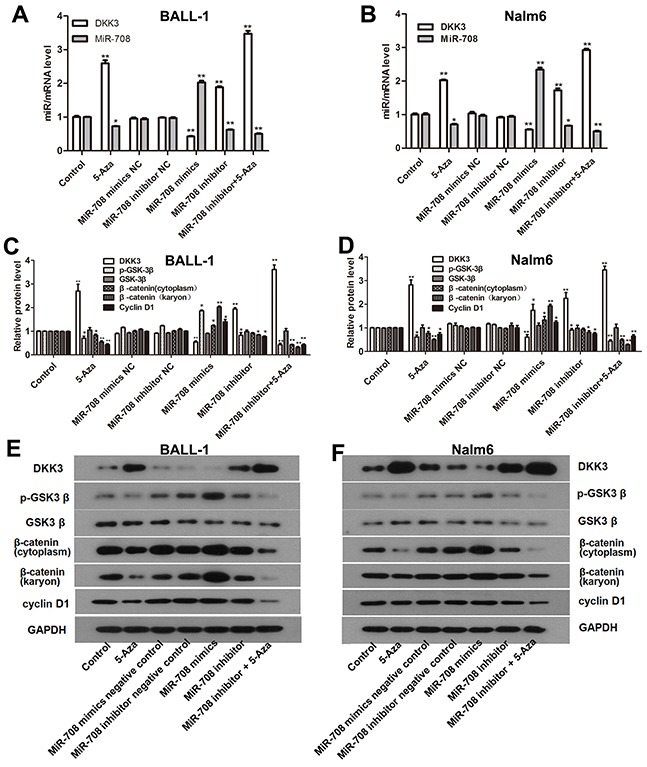
5-Aza and the miR-708 inhibitor increased DKK3 expression and inhibited the Wnt signaling pathway in the B-ALL cell lines **(A** and **B)**. DKK3 mRNA and miR-708 expression were altered when the cells were treated with 5-aza and the miR-708 inhibitor. **(C** and **D)** Expression of β-catenin (cytoplasmic and nuclear), cyclin D1, GSK3β and p-GSK3β in the B-ALL cell lines after treatment with 5-aza and the miR-708 inhibitor, as determined by western blotting. The graphs show the corresponding band intensities of β-catenin, cyclin D1, GSK3β and p-GSK3β normalized to GAPDH and compared with the control. **(E** and **F)**. The images represent β-catenin (cytoplasmic and nuclear), cyclin D1 GSK3β and p- GSK3β expression in BALL-1 cells after treatment with 5-aza and the miR-708 inhibitor/mimics or negative controls (NC), as determined by western blotting. The data are presented as the means ± SDs of triplicate experiments. *P<0.05; **P<0.01.

DKK3 is expressed at a low level that is related to the high miR-708 expression in adult B-ALL patients (Figure 1). The miR-708 inhibitor increased DKK3 mRNA and protein expression in the B-ALL cell lines (Figure 2). Because promoter methylation has been reported as another mechanism that leads to DKK3 down-regulation [11], we measured the promoter methylation state in BALL-1 and Nalm-6 cells after treatment with 5-aza by MSP. The DKK3 promoter was partially methylated in the BALL-1 and Nalm-6 cells. The unmethylated status of the DKK3 promoter increased significantly and the methylated status decreased after 5-aza treatment in both cell lines (Figure 6B). We also examined the effects of the miR-708 inhibitor and 5-aza on the CpG sites at the promoter region of the DKK3 gene by BSP. The Nalm-6 and BALL-1 cells exhibited DNA methylation at 43 CpG sites in the promoter region (Figure 6C and 6D). The low DKK3 expression level in these cells was confirmed by PCR (Figure 7A and 7B). After 5-aza treatment, DKK3 mRNA expression increased, and the sequence in this region was demethylated. Although the miR-708 inhibitor increased mRNA expression, it did not decrease the number of methylated CpG sites in the DKK3 promoter. When combined with 5-aza, the miR-708 inhibitor increased the number of unmethylated CpG sites, although the results were not significantly different than the results obtained with 5-aza treatment alone. Conversely, the miR-708 inhibitor and 5-aza sharply increased DKK3 mRNA expression compared with 5-aza treatment. Surprisingly, the miR-708 level was significantly decreased (Figure 7A and 7B) following 5-aza treatment.

### The miR-708 inhibitor and 5-aza suppress the Wnt/β-catenin pathway

DKK3 is a reported inhibitor of the Wnt/β-catenin signaling pathway [12]. To evaluate the effects of the miR-708 inhibitor and 5-aza on the Wnt signaling pathway, we examined the GSK3β, p- GSK3β, cyclin D1 and, cytoplasmic and nuclear β-catenin protein expression levels in the Nalm-6 and BALL-1 cells following treatment with these compounds. As shown in Figures 7C-7F, nuclear β-catenin expression was decreased following miR-708 inhibitor treatment, and this effect was significantly enhanced by 5-aza treatment in BALL-1 and Nalm-6 cells. Cytoplasmic β-catenin expression was slightly decreased by the miR-708 inhibitor, but the difference was not significant; however, after miR-708 inhibitor and 5-aza treatment, cytoplasmic β-catenin expression was significantly decreased (Figures 7C-7F). The p-GSK3β and cyclin D1 protein levels were also decreased upon miR-708 inhibitor treatment, and this effect was augmented by 5-aza (Figures 7C-7F), whereas GSK3β expression was not affected by miR-708 and/or 5-aza. These results suggest that the miR-708 inhibitor suppresses the Wnt/β-catenin signaling pathway and that this effect is enhanced when combined with 5-aza treatment. Additionally, this effect might largely result from the restoration of the DKK3 level.

## DISCUSSION

Decreased DKK3 expression is related to a poor prognosis in patients with ALL [11]. The low DKK3 expression level was believed to result from promoter methylation [11–14]. However, DKK3 mRNA expression was down-regulated in patients with unmethylated DKK3 promoters [11]. Consistent with previous studies, we also found that low DKK3 expression partially resulted from promoter hypermethylation in adult B-ALL. Additionally, our data demonstrated that suppression of DKK3 could be attributed to miR-708 up-regulation, revealing a new regulatory mechanism that contributes to DKK3 inactivation in adult B-ALL. Due to the strong correlation between high miR-708 levels and low DKK3 expression in the adult B-ALL samples evaluated in this study, aberrant miR-708 expression might serve as a novel mechanism for the activation of Wnt/β-catenin signaling in adult B-ALL, and this mechanism may be equally important for methylation in this context.

In the current study, we provided evidence that miR-708 plays an oncogenic role in B-ALL. However, miR-708 was previously shown to act as a tumor suppressor in hepatocellular carcinoma [23], ovarian cancer [29] and glioblastoma [30], and this miRNA also behaves as an oncomir in bladder cancer and lung adenocarcinoma [31, 32]. Taking these findings and our results into consideration, miR-708 appears to play a dual role as both a tumor-promoting and tumor-suppressing miRNA. Similar findings have been noted for other miRNAs, including miR-98, miR-186, and miR-375, underscoring the need to define the specific roles of certain miRNAs in different types of cancer. In B-ALL, miR-708 exerts an oncomir effect by inactivating the reported tumor suppressor gene DKK3.

DKK3 exhibits distinct expression patterns and produces specific effects on Wnt signaling compared with other DKK family members. DKK-1, -2 and -4 share the ability to inhibit the canonical Wnt/β-catenin pathway through binding to the LRP5/6 co-receptor with high affinity, but they are ineffective with respect to the non-canonical Wnt/PCP and Wnt/Ca^2+^ pathways [33, 34]. In contrast, the effects of DKK3 on the Wnt pathways are controversial. Our data indicate that DKK3 inhibits cell growth by suppressing the Wnt/β-catenin pathway in adult B-ALL patients. DKK3 was reported to selectively activate the c-Jun-NH_2_-kinase in human prostate cancer cells, suggesting inhibition of the non-canonical Wnt pathway [35]. In glioblastoma multiforme, DKK3 affects both the canonical and non-canonical Wnt signaling pathways [36]. Thus, the role of DKK3 in Wnt signaling might differ among tumor cell types, and investigating the precise Wnt pathways affected by DKK3 remains important. Glycogen synthase kinase 3β (GSK3β) is a known negative regulator of β-catenin. However, in our study, we found that DKK3 restoration decreases p-GSK3β and nuclear β-catenin expression in adult B-ALL. A recent study showed that spermidine/spermine N1-acetyltransferase (SSAT) overexpression inhibits β-catenin nuclear translocation and decreases p-GSK3β expression through AKT activation [37]. The *Pseudomonas aeruginosa* mannose-sensitive hemagglutinin (PA-MSHA) was reported to reduce β-catenin and p-GSK3β by affecting the PTEN/AKT signaling pathway [38]. Additionally, the neurokinin-1 receptor (NK-1R) activator increases β-catenin and p-GSK3β expression [39]. Based on these finding and our data, the reduction in β-catenin and p-GSK3β that occurs via the restoration of DKK3 might result from crosstalk with other signaling pathways.

Inactivation of DKK3 plays important roles in chemoresistance, which leads to the ineffectiveness of conventional chemotherapy and results in therapy failure [40]. Therefore, active silencing of DKK3 is a promising therapeutic strategy for tumors resistant to conventional chemotherapy [40]. Concordant with previous studies that have reported that the demethylating drug decitabine or Wnt inhibitor treatment reverses relapse-specific gene expression and restores chemosensitivity to significantly increase the treatment effects of ALL [41, 42], our data suggest that treatment with a miR-708 inhibitor and/or 5-aza might be a therapeutic strategy for adult B-ALL with DKK3 inactivation. Interestingly, our data show that 5-aza decreases miR-708 expression while increasing DKK3 expression, suggesting the presence of a feedback loop in the miR708/DKK3 pathway, as previously reported [43]. Resolution of this matter requires further investigation.

Another finding of this study is that miR-708 overexpression might potentially be used as a biomarker to predict relapses in adult B-ALL patients. These results correspond with a previous study suggesting that miR-708 can be used as a reliable indicator for the prediction of pediatric ALL relapses [24]. Additionally, elevated miR-708 expression might be related to a poor prognosis because both our results and those from a previous study have indicated that low DKK3 expression is correlated with a poor prognosis in ALL patients [11]. Another study also showed that the overexpression of miR-708 and down-regulation of its targets CNTFR and NNAT are related to high-risk common ALL in children and that miR-708 might play an important role in mediating the high relapse risk [25]. However, because the number of patients in our study was small, we could not address whether miR-708 had any predictive value for adult B-ALL. Further research with a larger cohort is needed to assess whether miR-708 is predictive of adult B-ALL relapse.

The present study provides insights into the mechanisms involved in leukogenesis and the progression of adult B-ALL. Our findings provide evidence that a miR-708 inhibitor elevates DKK3 expression independent of promoter demethylation and that restoration of DKK3 leads to suppression of the Wnt/β-catenin signaling pathway and cell growth. To the best of our knowledge, this study provides the first demonstration of a post-translational mechanism that regulates DKK3 expression in addition to promoter hypermethylation in adult B-ALL. Therefore, strategies for regulating miR-708 and/or DKK3 expression might offer potential for new therapeutic developments for B-ALL.

## MATERIALS AND METHODS

### Patient samples

We studied 37 patients (including 10 patients with AML (non-M3) and 27 patients with ALL) who were diagnosed with acute leukemia (according to the WHO diagnostic criteria [26]) at the Department of Hematology of the First Affiliated Hospital of Harbin Medical University (Harbin, Heilongjiang province, China). Approval was obtained from the Ethics Committee of Human Experimentation at Harbin Medical University. Informed consent was provided according to the current version of the Declaration of Helsinki. Bone marrow samples were collected, and mononuclear cells (MNCs) were harvested by Ficoll-Hypaque (Solarbio, Beijing, China) density gradient centrifugation. The median age at diagnosis for the study population as a whole was 35.45 years (range, 17-77 years, Supplementary Table 1). Ten patients were diagnosed with AML, 20 patients were diagnosed with B-ALL, and seven patients were diagnosed with T-ALL. Six patients with B-ALL (P13, 14, 16 and, 28-30) were also examined at the time of the initial diagnosis, when they achieved complete remission (CR) and during relapse. Single-cell suspensions obtained from bone marrow collected from healthy volunteers were stained for CD19 with a human-specific fluorescein isothiocyanate (FITC)-conjugated anti-CD19 antibody (Miltenyi Biotech, Bergisch Gladbach, Germany) according to the manufacturer's protocol. The stained cells were verified as CD19+ cells through fluorescence-activated cell sorting (FACS); the post-sorting analysis showed greater than 95% for the CD19+ cell population.

### Reagents

5-Aza-2′-deoxycytidine was purchased from Sigma (Sigma-Aldrich Corporation, St. Louis, MO, USA), dissolved in 100% DMSO to obtain a stock concentration of 10^−2^ M, stored at −20°C, and diluted to the desired concentrations in RPMI 1640 medium prior to use.

### Cell cultures

The human pre-B lymphocytic cell lines Nalm-6 and BALL-1 were kindly provided by the Shanghai Cell Bank of the Chinese Academy of Sciences (Shanghai, China). The cells were maintained in RPMI 1640 culture medium containing 10% fetal bovine serum (FBS), 100 U/mL penicillin and 100 U/mL streptomycin and incubated in a 5% CO_2_ humidified incubator at 37°C. The cells were treated with 10 μmol/L 5-aza for 48 h and/or a miR-708 inhibitor via transfection. Alternatively, the cells were transfected with a miR-708 mimic/inhibitor or co-transfected with a miR-708 inhibitor and DKK3-siRNA and then harvested.

### miRNA/siRNA transfection

The cells were plated in growth medium without antibiotics for approximately 24 h prior to transfection. Transient transfections of the miRNA precursor (Ambion)/siRNA (Origene/Invitrogen, USA) were performed using Lipofectamine 2000 (Invitrogen) according to the manufacturer's recommended protocol. The miRNA products used for transfection included a miR-708 mimic and a negative control miRNA mimic. The miR-708 mimics, miR-708 inhibitor and negative controls were purchased from GenePharma (Shanghai, China) as described previously [27]. The sequences were as follows: miR-708 inhibitor, CCC AGC UAG AUU GUA AGC UCC UU; miR-708 inhibitor negative control (NC), CAG UAC UUU UGU GUA GUA CAA; miR-708 mimics, AAG GAG CUU ACA AUC UAG CUG GG and CAG CUA GAU UGU AAG CUC CUU UU; and miR-708 mimic NCs, UUC UCC GAA CGU GUC ACG UTT and ACG UGA CAC GUU CGG AGA ATT. The mature type of short-interfering RNA (siRNA) for DKK-3 (siR-DKK-3; Invitrogen) was used for the transfection of the cells. The sequence of siR-DKK3 were 5′-GAUGAGUAUGAAGUUGGCAGCUUCA-3′ and 5′-CCCTCTTTGGCAGTTGCATTAGTAA-3′. The seq uence of non specific control siRNA was : 5′-UUCUCCG AACGUGUCACGUTT-3′. The cells were harvested at different time points.

### Predicting the regulatory miRNA of DKK3

miRanda (http://cbio.mskcc.org/miRNAviewer/, Memorial Sloan-Kettering Cancer Center) and miRBase (http://www.mirbase.org) were used to predict potential regulatory miRNAs targeting the DKK3 gene.

### Plasmid construction and luciferase assay

To construct the luciferase reporter plasmid, a fragment of DKK3 was inserted into the psiCHECK™-2 vector (Promega, WI, USA). Cells were seeded into 24-well plates at a density of 2×10^4^ cells/well and maintained in RPMI 1640 culture medium containing 10% FBS. The next day, the culture medium was replaced with 300 μL of Opti-MEM. For each well, 1 μL of the miR-708 mimic (20 μM) or inhibitor was co-transfected with 0.5 μg of the luciferase reporter plasmid using the Lipofectamine 2000 transfection reagent according to the manufacturer's instructions (Invitrogen, Grand Island, NY, USA). Forty-eight hours after transfection, the luciferase activity was measured using a Dual-Luciferase Reporter Assay System (Promega), and the relative luciferase intensity was determined.

### Cell proliferation analysis

To evaluate cell proliferation, cells were seeded into a 96-well plate at a density of 1×10^4^ cells/well. A total of 10 μL of Cell Counting Kit-8 (CCK-8; Sigma-Aldrich) solution was added to 100 μL of culture medium. After the cells were incubated for 4 h at 37°C, the absorbance of the culture medium was measured at 450 nm (A450) using a scanning microplate spectrophotometer (Multiscan MK3, Thermo Fisher Scientific).

### Cell cycle and apoptosis analysis

For the cell cycle analysis, the cells were fixed overnight in chilled methanol before staining with 50 μg/mL propidium iodide (PI, Sigma-Aldrich) in the presence of 1 mg/mL RNase (100 units/mL; Sigma-Aldrich) and 0.1% NP40 (Sigma-Aldrich).

For the apoptosis analysis, the samples were incubated with Annexin V- FITC/PI according to the manufacturer's recommended protocol (Sigma-Aldrich). Cell-bound fluorescence was analyzed using a FACSCalibur flow cytometer (Becton Dickinson, CA, USA).

### RNA extraction and quantitative real-time PCR (qRT-PCR)

Total RNA was extracted using the TRIzol reagent (Invitrogen). Then, 1 μg of the total RNA was reverse-transcribed, and qRT-PCR was performed using the ABI PRISM 7900 sequence detection system (Biosystems). The PCR amplification was performed in a reaction system containing cDNA, forward and reverse primers, 2X SYBR Green qPCR SuperMix, and distilled water. DKK3 transcript expression was measured by qRT-PCR using the forward primer 5′-TTTTCCACGCAGTTCTTTCC-3′ and the reverse primer 5′-TGAGCCTCTGAGATCCCTGA-3′. For the DKK3 transcript expression measurements, β*-*actin was amplified as an internal control using the forward primer 5′-CTTAGTTGCGTTACACCCTTTCTTG-3′ and the reverse primer 5′-CTGTCACCTTCACCG TTCCAGTTT-3′. The relative expression of the amplified RNA samples was calculated using the 2^−DDCT^ method. The results are presented as the fold change of each mRNA relative to a control sample (MNCs collected from a healthy volunteer).

### miRNA real-time PCR (miR-qRT-PCR)

miRNAs were isolated using a miRNeasy Mini Kit (Qiagen, Hilden, Germany) and reverse transcribed with miRNA-specific stem loop RT primers (Life Technologies, Grand Island, NY, USA) for 30 min at 16°C, 30 min at 42°C, and 5 min at 85°C. Real-time PCR was performed in duplicate using the following conditions: 95°C for 10 min, followed by 40 cycles of 95°C for 15 s and 60°C for 1 min. The primer sequences of miR-708 were as follows: forward, 5′-CCGCACGAAGGAGCTTACAAT-3′; and reverse, 5′-GTGCAGGGTCCGAGGTATTC-3′. Each miRNA expression value is represented relative to the expression of the U6 small nuclear RNA (snRNA), which was amplified as an internal control using the U6 forward primer 5′-CTCGCTTCGGCAGCACA-3′ and reverse primer 5′-AACGCTTCACGAATTTGCGT-3′. The expression level of miR-708 was determined using the 2^−ΔΔCT^ method. The results are presented as the fold changes of each miRNA relative to a control sample (MNCs collected from a healthy volunteer).

### DNA extraction and methylation-specific PCR (MSP)

Cells were collected following 5-aza (10 μM) treatment as described previously [28]. DNA was extracted and bisulfite-converted using an EZ DNA Methylation-Gold Kit (ZYMO Research, Foster City, CA, USA). All bisulfate-treated DNA samples (1 μg) were amplified using primers specific for the methylated or unmethylated sequence. For the unmethylated reaction, the DKK3 forward primer 5′-TTTTGGTTTTTTTTTGTTTTTGGG-3′ and the reverse primer 5′-CCAAACCACTACATCTCCACT-3′ were used, whereas for the methylated reaction, the DKK3 forward primer 5′-CGGTTTTTTTTCG TTTTCGGG-3′ and the reverse primer 5′-CAAACCGCTA CATCTCCGCT-3′ were used. PCR was performed using a GeneAmp DNA Amplification Kit and AmpliTaq Gold Polymerase (Perkin Elmer, Foster City, CA, USA) with the following conditions: 95°C for 5 min followed by 35 cycles of 95°C for 30 s, 60°C for 30 s, and 72°C for 40 s. A total of 10 μL of the PCR product was separated by 2.5% agarose gel electrophoresis and stained with the GoldView I nucleic acid stain for 45 min, and the results were photographed and analyzed.

### BSP

The bisulfite sequencing PCR (BSP) reaction system was composed of 1X PCR buffer (0.25 mM KCl), 6.25 μM of a dNTP mixture, 0.5 μM of the primers, 0.75 U of the hot start DNA polymerase (TaKaRa, Tokyo, Japan), and 20 ng of the modified DNA (bisulfite-converted as described above). First-roud PCR consisted of 35 cycles at an annealing temperature of 56°C, with the use of the primers MS1 (5′-GGAGTTGAATTTCGGAAGAT-3′) and MS2 (5′-TCCTCCATCAATTCCTCAACC-3′) (Figure 6A). With the use of the twentieth of the first-round PCR product as a template, second-round PCR consisted of 35 cycles at an annealing temperature of 60°C with the primers MS3 (5′-TTCGGGTGTAGGGGAGTTG-3′) and MS4 (5′-TCTCATTAAAAATAACCTCCTCC-3′) (Figure 6A). Under these conditions, we could assess the DNA methylation status of the DKK3 gene, which had a length of 342 bp and contained 43 CpG sites upstream of the transcription initiation site. The PCR products were analyzed on a 1.5% agarose gel. Each purified product was cloned into the pMD19-T Vector (TaKaRa) and transfected into DH5α competent cells (Vazyme Biotech Co., Piscataway, NJ, USA). Five to 10 clones from each sample were subjected to cycle sequencing (PE Applied Biosystems, Warrington, UK) and analyzed using the ABI 310 sequencer (Applied Biosystems, Foster City, CA, USA).

### Western blotting analysis

Proteins were extracted from the cells, separated by SDS-PAGE and transferred onto polyvinylidene fluoride membranes. The membranes were blocked with 5% non-fat dry milk and incubated overnight at 4°C with primary antibodies against the following proteins: GSK3β, p-GSK3β (Ser 9), DKK3, β-catenin, cyclin D1, Bcl-2, and Bax-1. These antibodies were purchased from Abcam (Shanghai, China). An anti-GAPDH or β-actin antibody obtained from Sigma-Aldrich was used as a loading control. The resulting bands on the immunoblots were visualized using a BCIP/NBT kit (Sigma, St. Louis, MO, USA). The band intensities from the western blotting experiments were quantified with image analysis software (ImageQuant TL; Amersham Biosciences) prior to the statistical analysis.

### Statistical analysis

All results were obtained from at least three separate experiments. The data are expressed as the means ± SD. SPSS 17.0 was used for the statistical analyses. The statistical comparisons were performed using one-way analysis of variance and Student's t-test. Differences were considered significant at *P*<0.05. Two-tailed tests were used for the univariate comparisons. The correlation analysis between DKK3 mRNA and miR-708 expression was performed through Spearman's correlation analysis.

## SUPPLEMENTARY MATERIALS TABLE



## Abbreviations

DKK3: Dickkopf-3
B-ALL: B acute lymphoblastic leukemia
5-aza: 5-aza-2′-deoxycytidine
MSP: methylation-specific PCR
BSP: bisulfite genomic sequencing PCR
LEF/TCF: lymphoid enhancer factor/T cell factor
AML: acute myelocytic leukemia
miRNAs: microRNAs
UTR: untranslated region
GSK3β: glycogen synthase kinase 3β
SSAT: spermidine/spermine N1-acetyltransferase
PA-MSHA: *Pseudomonas aeruginosa* mannose-sensitive hemagglutinin
NK-1R: neurokinin-1 receptor
