# Supplementary material for: Suppression of miR-708 inhibits the Wnt/β-catenin signaling pathway by activating DKK3 in adult B-all
Source: Oncotarget. 2017 Jul 18;8(38):64114–28. doi: 10.18632/oncotarget.19342 (PMC5609988; doi:10.18632/oncotarget.19342)
Supplement: Supplementary file 1 [file oncotarget-08-64114-s001.pdf]

Suppression of miR-708 inhibits the Wnt/β-catenin signaling pathway by activating DKK3 in adult B-all

SUPPLEMENTARY MATERIALS

Supplementary Table 1: Patient characteristics

See Supplemantary File 1
